# The use of a *P. falciparum* specific coiled-coil domain to construct a self-assembling protein nanoparticle vaccine to prevent malaria

**DOI:** 10.1186/s12951-017-0295-0

**Published:** 2017-09-06

**Authors:** Christopher P. Karch, Tais A. P. F. Doll, Sara M. Paulillo, Issa Nebie, David E. Lanar, Giampietro Corradin, Peter Burkhard

**Affiliations:** 1 Institute of Materials Science, University of Connecticut, Storrs, CT 06269-3136 USA; 2 Alpha-O Peptides AG, 4125 Riehen, Switzerland; 3 Centre National de Recherche et de Formation sur le Paludisme, 01 BP 2208 Ouagadougou, West Africa Burkina Faso; 4 Malaria Vaccine Branch, USMMRP-WRAIR, Walter Reed Army Institute of Research, Silver Spring, MD 20910 USA; 5 Biochemistry Department, University of Lausanne, 1066 Epalinges, Switzerland; 6 Department of Molecular and Cell Biology, University of Connecticut, Storrs, CT 06269-3125 USA

**Keywords:** Self-assembly, Protein nanoparticles, Malaria, Vaccine, P27, P27A, Tex1, Blood stage

## Abstract

**Background:**

The parasitic disease malaria remains a major global public health concern and no truly effective vaccine exists. One approach to the development of a malaria vaccine is to target the asexual blood stage that results in clinical symptoms. Most attempts have failed. New antigens such as P27A and P27 have emerged as potential new vaccine candidates. Multiple studies have demonstrated that antigens are more immunogenic and are better correlated with protection when presented on particulate delivery systems. One such particulate delivery system is the self-assembling protein nanoparticle (SAPN) that relies on coiled-coil domains of proteins to form stable nanoparticles. In the past we have used de novo designed amino acid domains to drive the formation of the coiled-coil scaffolds which present the antigenic epitopes on the particle surface.

**Results:**

Here we use naturally occurring domains found in the tex1 protein to form the coiled-coil scaffolding of the nanoparticle. Thus, by engineering P27A and a new extended form of the coiled-coil domain P27 onto the N and C terminus of the SAPN protein monomer we have developed a particulate delivery system that effectively displays both antigens on a single particle that uses malaria tex1 sequences to form the nanoparticle scaffold. These particles are immunogenic in a murine model and induce immune responses similar to the ones observed in seropositive individuals in malaria endemic regions.

**Conclusions:**

We demonstrate that our P27/P27A-SAPNs induce an immune response akin to the one in seropositive individuals in Burkina Faso. Since P27 is highly conserved among different *Plasmodium* species, these novel SAPNs may even provide cross-protection between *Plasmodium falciparum* and *Plasmodium vivax* the two major human malaria pathogens. As the SAPNs are also easy to manufacture and store they can be delivered to the population in need without complication thus providing a low cost malaria vaccine.

## Background

Malaria, the mosquito-borne parasitic disease caused by members of the *Plasmodium* genus, resulted in at least 438,000 deaths worldwide in 2014 [1]. Currently, there is an attempt to eliminate the disease from the human population. Attempts at vector control, diagnosis, and development of pharmaceuticals have all been successful in the reducing the number of cases, however, a major missing piece in the elimination campaign is an effective vaccine candidate. The current most successful vaccine candidate RTS,S is only about 30% protective in children in Sub-Saharan Africa, the most vulnerable population [2, 3]. The goal of malaria elimination and the weaknesses of RTS,S necessitate the development of new more successful vaccine candidates.

One of the most appealing, yet elusive, malaria vaccine candidates is one targeting the asexual blood stage, the clinical stage of the disease. Issues such as antigen polymorphism, lack of MHC I molecules on erythrocytes, and speed of erythrocyte infection all hamper the development of an asexual blood stage vaccine [4–9]. Many different antigen candidates and technical approaches have been attempted with little to no success. These problems have led to the search for new potential blood stage antigens using different screening mechanisms including bioinformatic approaches.

In one such bioinformatic approach coiled-coil domains present in the asexual blood stage were targeted [10]. Coiled-coil domains are a common oligomerization domain in proteins known for their characteristic heptad repeat and stability, making them excellent choices for vaccine development [11]. One of the most interesting targets identified in this screen was P27, which showed complete conservation in field isolates [12]. P27 is a 27 amino acid sequence contained on the 1103 amino acid trophozoite export protein (Tex1) [13]. However, P27 was only able to activate 30% of peripheral blood mononuclear cells (PBMC) from semi-immune individuals, leading to fears that it would not offer broad protection in a vaccinated diverse population. This lead to further screening of the Tex1 protein and the identification of P27A in the N-terminal portion of the protein, a region that was predicted to be intrinsically unstructured. Initial screenings demonstrated that people living in endemic areas had antibodies for P27A, it was immunogenic in small mammal models, and antibodies raised against P27A were capable of inhibiting *P. falciparum* in antibody-dependent cellular inhibition parasite-growth assay [14]. Currently, good immunogenicity results were obtained in phase Ia and Ib clinical trials of P27A combined with the adjuvants GLA-SE or Alhydrogel (ClinicalTrials.gov; PACTR201310000683408; manuscript in preparation).

Subunit vaccines based solely on recombinant proteins are generally weakly immunogenic and will most likely not be successful for vaccination in humans. To be an effective vaccine an important consideration is the delivery system. Several recent effective vaccine candidates are based on particulate delivery systems that are able to repetitively express antigens [15]. One promising delivery system is the self-assembling protein nanoparticle (SAPN) [16]. Each SAPN monomer, the subunit that oligomerizes to form the nanoparticle, contains two coiled-coil domains held together by a linker. Antigenic B-cell epitopes are engineered on either the N or C terminal ends of the gene encoding the monomer. In the same way T-cell epitopes can be added to the core leading to a vaccine candidate that is able to activate both the humoral and cellular branches of the immune system. SAPNs show great promise for vaccine design [15, 17–19] and many SAPN-based malaria vaccines have previously been developed in our laboratories [20–23].

Normally, the sequence of amino acids that form the pentameric and trimeric oligomerization domains are de novo designed sequences that do not have homology to any human proteins to minimize the possibility of inducing an immune response that could be detrimental to the host receiving the vaccine. Here we detail the unusual step in the development of a SAPN vaccine candidate by using a parasite native coiled-coil sequence, the P27 epitope from the Tex1 protein, to form part of the core oligomerization domains. In combination with the intrinsically unstructured epitope P27A, this vaccine candidate is immunogenic in a murine model and induces antibodies that are recognizing the same antigens as sera from seropositive individuals in Burkina Faso, a malaria endemic region.

## Methods

### Peptide sequences

- Pept-P27-N (845–871):
- KKRNVEEELHSLRKNYNIINEEIEEIT
- Pept-P27A (223–326):
- HNNNEKNISYDKNLVKQENDNKDEARGNDNMCGNYDIHNERGEMLDKGKSYSGDEKINTSDN AKSCSGDEKVITSDNGKSYDYVKNESEEQEEKENMLNNKKRS
- Pept-P27-NC (846–893):
- KRNVEEELHSLRKNYNIINEEIEEITKEFEKKQEQVDEMILQIKNKELE
- Pept-P27-C (872–893):
- KEFEKKQEQVDEMILQIKNKELE

### Peptide synthesis

The single antigens *Pept*-*P27*-*N, Pept*-*P27A, Pept*-*P27*-*NC* and *Pept*-*P27*-*C* present in the SAPN scaffold were synthesized by solid-phase Fmoc chemistry using Applied Biosystems 431A and 433A synthesizers (Foster City, CA) as previously described [24]. Peptide purity was assessed by analytic C18 HPLC and mass spectrometry (MALDI-TOF, Applied Biosystems) and was higher than 80%. All the reagents used were purchased from Fluka (Buchs, Switzerland) or Novabiochem (Läufelfingen, Switzerland).

### SAPN protein sequences

- SAPN-P27-1:
- MGHHHHHHASGSWEEWNAKWDEWIRAWVAWRAAWEKWKDDWAYWTLTWKYGELYSKLRNVEEELHSLRKNYNIINEEIEEITKEFEKKQEQVDEMIIQIKNKELE
- SAPN-P27-2:
- MGHHHHHHASLIDYNKAALSKFKERGSWQTWNAKWDVWSNDWNAWRARWQAWVDDWAYWTLTWKYGELYSKLRNVEEELHSLRKNYNIINEEIEEITKEFEKKQEQVDEMIIQIKNKELE
- SAPN-P27A-1:
- MGHHHHHHASGSWQTWNAKWDVWSNDWNAWRADWQAWVDDWAYWTLTWKYGELYSKLAEIERRVEANERALEELAQFVRALSMQAAELERRIEEIARGHNNNEKNISYDKNLVKQENDNKDEARGNDNMSGNYDIHNERGEMLDKGKSYSGDEKINTSDNAKSSSGDEKVITSDNGKSYDYVKNESEEQEEKENMLNNKKRS
- SAPN-P27A-2:
- MGHHHHHHASYYIPHQSSLPGSWQTWNAKWDVWSNDWNAWRADWQAWVDDWAYWTLTWKYGELYSKLAEIERRVEANERALEELAQFVRALSMQAAELSEDITKYFRHILYISFYFILVNRARGHNNNEKNISYDKNLVKQENDNKDEARGNDNMSGNYDIHNERGEMLDKGKSYSGDEKINTSDNAKSSSGDEKVITSDNGKSYDYVKNESEEQEEKENMLNNKKRS
- SAPN-Combo:
- MGDHHHHHHHHHHAAHAAHAAHAAHAAAARGHNNNEKNISYDKNLVKQENDNKDEARGNDNMCGNYDIHNERGEMLDKGKSYSGDEKINTSDNAKSCSGDEKVITSDNGKSYDYVKNESEEQEEKENMLNNKKRSASGSAKFVAAWTLKAAASGSWEEWNAKWDEWRNDQNDWREDWQAWRDDWAYWTLTWRYGELYSRLARIERRVEELRRLLQLIRHENRMVLQFVRALSMQARRLESKLRNVEEELHSLRKNYNIINEEIEEITKEFEKKQEQVDEMILQIKNKELE

### Gene synthesis

Codon optimized genes for *E. coli* were synthesized by GeneScript USA Inc. (Piscatway, NJ) containing the sequence of the constructs *SAPN*-*P27*-*1, SAPN*-*P27*-*2, SAPN*-*P27A*-*1, SAPN*-*P27A*-*2* and *SAPN*-*Combo*. Each gene was cloned into a pPEP-T vector. The resulting constructs were transformed into Tuner (DE3) Competent Cells (Millipore, Billerica, MA).

### Protein expression

Protein expression was carried out in Luria–Bertani (LB) Broth (FisherBioReagents, Pittsburgh, PA). Briefly, starter cultures were grown for 16 h at 36 °C and inoculated at 1/100 into 1L of LB and grown at 37 °C. At an OD_600_ of 0.8 cultures were induced with 1 mM Isopropyl-β-d-thiogalactopyranoside (IPTG) (FisherBioReagents, Pittsburgh, PA). Cultures were grown for 4 h, pelleted at 4000× g and stored at −80 ºC until use.

### Protein purification

Cell pellets were thawed on ice and resuspended in Imidazole free buffer (8 M urea, 100 mM NaH_2_PO_4_, 20 mM Tris base, 5 mM tris(2-carboxyethyl)phosphine, pH 8.0), lysed by sonication, and centrifuged at 30,500× *g* for 25 min to clarify the lysate. Purification was carried out on an ÄKTApurifier 100 (GE Healthcare, Piscataway, NJ) using a 5 mL HisTrap HP column (GE Healthcare, Piscataway, NJ). Briefly columns were equilibrated with Binding Buffer (8 M urea, 100 mM NaH_2_PO_4_, 20 mM Imidazole, 20 mM Tris base, 5 mM tris(2-carboxyethyl)phosphine, pH 8.0), lysate injected, and then washed with five column volumes of binding buffer. Columns were then washed with 5 column volumes of high phosphate buffer (8 M Urea, 500 mM NaH_2_PO_4_, 20 mM Tris base, 5 mM tris(2-carboxyethyl)phosphine, pH 8.0), binding buffer, isopropanol wash (60% isopropanol, 20 mM Tris base, pH 8.0) to remove LPS [25], and finally Imidazole free buffer. Protein was eluted using a 1M Imidazole gradient. The resulting recovered protein was verified by a SDS-PAGE, and dialyzed overnight into pre-refolding buffer (8 M urea, 50 mM NaCl, 20 mM Tris, 5% Glycerol, tris(2-carboxyethyl)phosphine, pH 8.0).

### SAPN refolding

SAPNs were then allowed to refold in a stepwise manner, slowly removing urea and tris(2-carboxyethyl)phosphine and resulting in refolded SAPN in 20 mM Tris base, 5% glycerol, pH 8.5.

### DLs

Refolding was monitored by dynamic light scattering using a Malvern Zetasizer Nano S (Malvern, Worcestershire, UK) equipped with a 633-nm laser. The hydrodynamic diameter was determined at 25 °C. Each sample was run 5 times, and the average result taken.

### Transmission electron microscopy

0.025 mg/mL of each sample was adsorbed onto carbon coated grids (Electron Microscopy Sciences Inc., Hatfield, PA) that were subjected to a 10 s glow discharge, washed with distilled water three times, and negatively stained with 0.5% uranyl acetate (Electron Microscopy Sciences Inc., Hatfield, PA). Electron micrographs were taken on an FEI Tecnai T12 Transmission Electron Microscope.

### Human plasma

Human plasma were collected during the malaria transmission season from adult donors living in Burkina Faso. Plasma from Swiss naive donors who had no malaria history were pooled and used as a negative control. Blood was taken by venipuncture into tubes containing EDTA according to the ethical clearance.

### Immunization

BALB/c and C3H mice (5/group) were immunized with 10 μg of NP in PBS at the base of the tail [26] three times at 3 weeks interval. Mice were individually bled 10 days after the 2nd and 3rd immunization from the tail vein and corresponding sera were stored at −20 °C. Mouse studies were conducted according to the protocol VD 805.9 approved by the state committee for animal research.

### Enzyme-linked immunosorbent assay (ELISA)

Prevalence (in humans) and titers (in mice) were determined by ELISA; antigens were diluted in PBS and used to coat ELISA plates. Antigen concentration for coating the 96-well flat plates (BD Biosciences, Allschwil, Switzerland) was 1 μg/ml (50 μl) for peptides longer than 40 residues, and 5 μg/ml for peptides shorter or equal to 40 residues as previously described [16, 17]. Secondary Ab, (sheep or goat anti-human or mouse polyvalent immunoglobulin (G, A, M)-AP (Sigma-Aldrich, St Louis, MO, 50 µl/well) was used at dilution indicated by the provider in PBS-T plus 2.5% milk. Prevalence was determined at plasma dilution of 1:200. Prevalence is positive when OD sample is ≥OD control + 3SD). Titer is defined as the last dilution where OD sample is ≥OD control + 3SD.

### Competitive inhibition ELISA

ELISA plates were coated with the appropriate peptide as described above, washed and blocked. Serum samples at appropriate dilutions (at about 50–60% of maximum OD as determined by ELISA) were pre-incubated with appropriate competing synthetic peptides at increasing concentrations for 30 min at room temperature. 50 μl of the antibody-peptide mixture was added into the appropriate wells and incubated for 1 h at room temperature in a humid chamber. ELISA was then completed as described above.

## Results

### SAPN design

We have already shown that SAPNs are an effective carrier for the pre-erythrocytic malaria antigen Circumsporozoite Protein (CSP) [20–23]. We believe the SAPN technology will be an excellent platform for the development of a malaria vaccine candidate based on the previously studied epitopes from the Tex1 protein. The prototypical SAPN design consists of a pentameric coiled-coil oligomerization domain linked to a trimeric coiled-coil oligomerization domain [16]. P27 has been found by prediction of its trimeric coiled-coil within the 27 residues. However, according to the coiled-coil prediction program PCOILS, the coiled-coil sequence extends over a span of at least 48 residues (Fig. 5). This prompted us to engineer a longer sequence of 50 residues containing the N-terminal P27-N and the C-terminal P27-C sequences. The whole extended coiled-coil stretch is then called P27-NC. Remarkably, the C-terminal fragment P27-C is highly conserved among many *Plasmodium* species (Fig. 5, and [14]) and thus potentially providing a cross-protecting immune response. The SAPNs’ unique design allows P27-NC to be stably displayed in its native coiled-coil conformation. This should increase its immunogenicity and specificity and thus create a more effective vaccine candidate. Such an approach has previously been taken for the trimeric coiled-coil epitope of the SARS spike protein [29]. P27A is an intrinsically unstructured epitope, so it does not have the same conformation restrictions as a domain with a ridged shape.

Each pentameric coiled-coiled domain will self-assemble with four other pentameric coiled-coiled domains, while each trimeric coiled-coiled domain will self-assemble with two other trimeric coiled-coil domains. To satisfy all of the assembly requirements 15 monomers will self-assemble into a structure known as a least common multiple unit (LCM). In a standard icosahedral structure four LCMs can further self-assemble into a full SAPN structure, meaning that 60 monomers make up each SAPN. However, recent biophysical studies combined with an analysis using viral tiling theory has shown that the SAPNs can possibly also assemble into higher order assemblies containing multiples of 60 chains per particle [27]. Each SAPN is predicted to contain 60 copies (or a multiple thereof) of P27A and P27-NC epitopes (Fig. 2b, d), indicating that they are effective at repetitively displaying each epitope, which is known to result in a more effective immune response.

Another advantage of the SAPN design is that within the SAPN core universal T-helper epitopes can be engineered that ideally lead to more effective immune response. These epitopes range from de novo designed sequences that are engineered to bind to a wide range of MHC II haplotypes to sequences from known infectious agents that have been established to result as strong T-helper epitopes [28–37]. Together, the repetitive display of both epitopes (P27-NC and P27A) combined with the addition of the universal CD4 epitopes should lead to a broadly immunogenic vaccine candidate that is able to protect a diverse population of humans against *P. falciparum*.

**Fig. 1:**
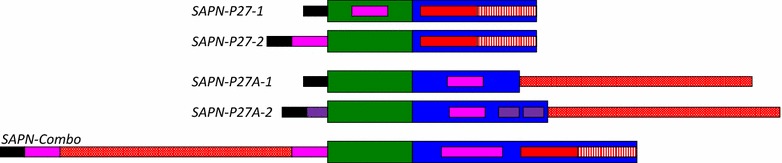
*From top to bottom* the schematic drawing of the constructs *SAPN*-*P27*-*1, SAPN*-*P27*-*2, SAPN*-*P27A*-*1, SAPN*-*P27A*-*2* and *SAPN*-*Combo* are shown. Coiled coils are represented by *thick boxes* with the pentamer in *green* and the trimer in *blue* color. The non-coiled-coil structures are shown in *thin boxes*. The color coding is as follows: B-cell epitopes—*red*; CD4-epitopes—*magenta*; CD8-epitopes—*purple*; His-tag—*black*. The P27 B-cell epitope (*red*; N-terminal [P27-N] *part solid*; C-terminal [P27-C] *part striped*) is part of the trimeric coiled coil while the unstructured P27A (*red* with *white* dots) is attached at either the C-terminal or the N-terminal end of the coiled coil

**Fig. 2:**
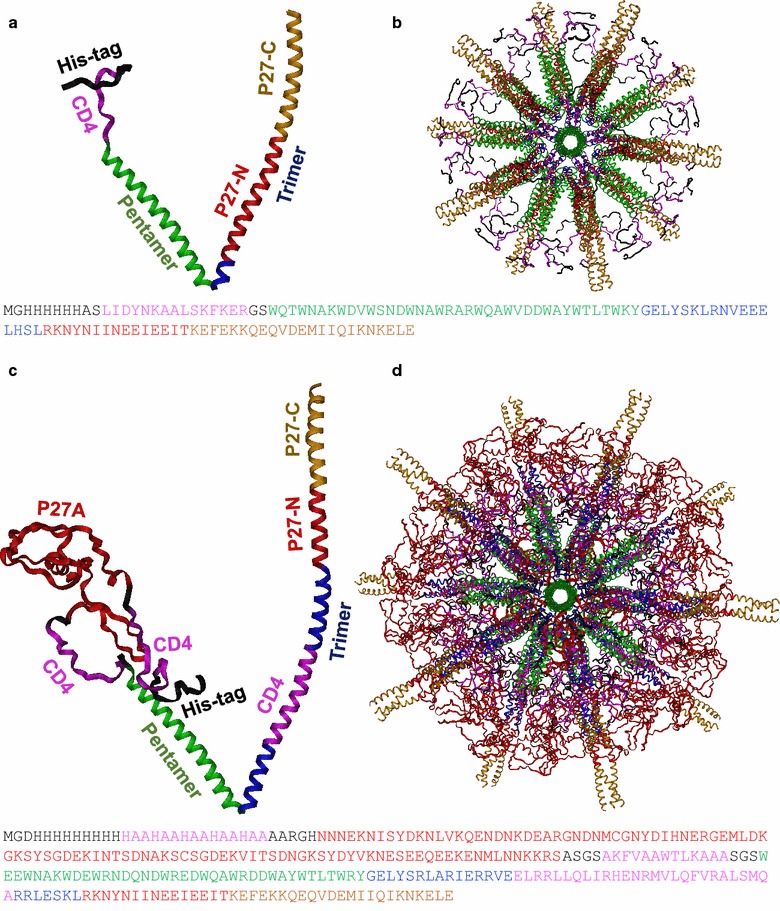
Molecular models of the *SAPN*-*P27*-*2* (*top*) and *SAPN*-*Combo* (*bottom*) assuming icosahedral symmetry. The view is down the fivefold symmetry axis. **a**, **c** Molecular models of the monomers; **b**, **d** molecular models of the particle. The color coding of the models is indicated in the sequences of the protein chains below. Monomers and particles are both drawn to size between *SAPN*-*P27*-*2* and *SAPN*-*Combo.* The epitope P27-C is clearly more exposed within the particle than the epitope P27-N, i.e. access to P27-N is more restricted

**Fig. 3:**
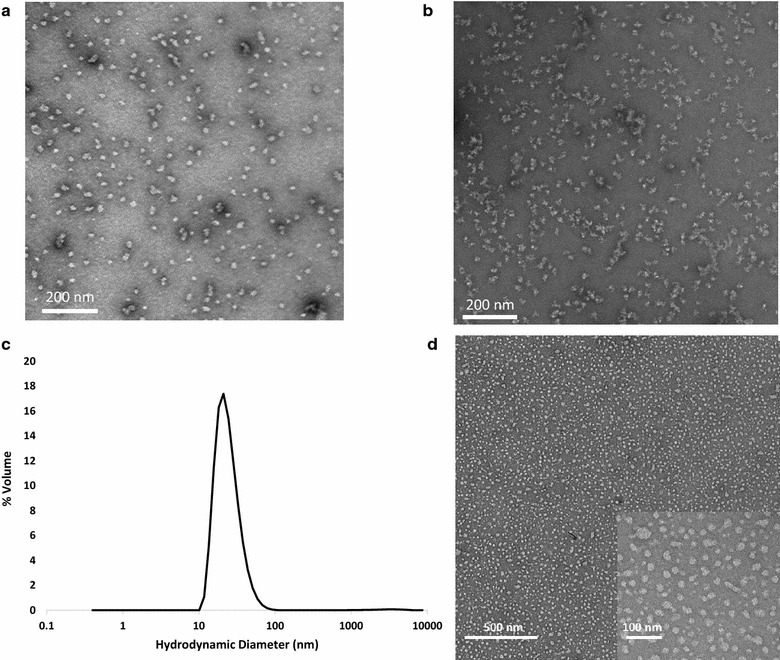
**a** TEM micrograph of *SAPN*-*P27A*-*1*; **b** TEM micrograph of *SAPN*-*P27A*-*2*; **c** DLS scan of the *SAPN*-*Combo*; **d** TEM micrograph of the *SAPN*-*Combo* with an *inset* at higher resolution

We engineered several SAPNs containing either the extended, 50 residue-long P27-NC trimeric coiled coil and/or the P27A intrinsically unstructured domain. A schematic representation of the five main constructs *SAPN*-*P27*-*1, SAPN*-*P27*-*2, SAPN*-*P27A*-*1, SAPN*-*P27A*-*2* and *SAPN*-*Combo* is shown in Fig. 1 and the corresponding protein sequences are listed in “Methods” section. Computational models of *SAPN*-*P27*-*2* and *SAPN*-*Combo* are displayed in Fig. 2. While the constructs containing only the trimeric coiled-coil epitope P27-NC as a B-cell epitope (*SAPN*-*P27*-*1* and *SAPN*-*P27*-*2*) lead to severe aggregation, the constructs with the intrinsically unstructured epitope P27A (*SAPN*-*P27A*-*1* and *SAPN*-*P27A*-*2*) formed very nice and soluble nanoparticles (Fig. 3a, b). *SAPN*-*P27A*-*1* was characterized in 50 and 150 mM NaCl in a 20 mM phosphate buffer at the pH 6.8 and 7.4 and 5% glycerol. All four conditions resulted in satisfactory nanoparticle formation. This construct was further characterized by circular dichroism for evaluation of secondary structure and mainly ∝-helical conformation was confirmed. Prior to circular dichroism analysis, a DLS run was taken to ensure nanoparticles were within expected range. For the construct *SAPN*-*P27A*-*2* even the incorporation of additional features such as three CD8 epitope strings predicted by NetMHC (YYIPHQSSL, ELSEDITKY and HILYISFYFILV—see also reference [38]) from the liver stage antigen 1 (LSA1) of the strain *P. falciparum* 3D7 in allowed formation of nice nanoparticles (Fig. 3b). We then reasoned that combining the problematic P27-NC epitope with the well-behaved P27A epitope in one single construct might help formation of well-shaped nanoparticles that contain the trimeric coiled-coil epitope P27-NC. Indeed, the *SAPN*-*Combo* construct with both epitopes was structurally and biophysically well-behaved (Fig. 3c, d). Thus, in such a construct we would be able to combine both Tex1 derived B cell epitopes in one single protein chain forming a SAPN.

### Production and refolding of *SAPN*-*Combo*

SAPN protein monomers were expressed in Tuner (DE3) *E. coli* and purified under denaturing and reducing conditions using immobilized metal chromatography. Resulting protein was run on SDS-Page gel to verify that the correct protein was purified. The primary product was in the expected size range of 34.3 kDa.

SAPN monomers were allowed to refold by slowly removing the denaturant and the reducing agent. The resulting product was screened by DLS to determine particle size. Based upon the DLS readings the refolded *SAPN*-*Combo* has a hydrodynamic diameter of 26.4 nm, which falls into the expected range for refolded SAPNs (Fig. 3c). The results of the DLS were further validated by TEM, which show *SAPN*-*Combo* has taken on particulate structure (Fig. 3d). These results demonstrate that we have successfully expressed and refolded the *SAPN*-*Combo*.

### Antigenicity and immunogenicity

The prevalence of the human antibody response specific to the *SAPN*-*Combo* construct and its single components was determined by measuring the percentage of adult responders living in Burkina Faso (Table 1). This response is similar to that originally obtained for the individual components *Pept*-*P27*-*N* and *Pept*-*P27A* (55 and 76% with mean OD of 0.26 and 0.67, respectively) in terms of % prevalence and average OD [10, 14].

**Table 1:** Prevalence in Burkina-Faso

Antigen	Mean OD	% positive donors
*SAPN*-*Combo*	0.62	66
Pept-P27-NC*	0.43	62
Pept-P27-C*	0.22	62

Sera of 48 donors leaving in Burkina-Faso were tested in ELISA at serum dilution of 1:200

**Fig. 4:**
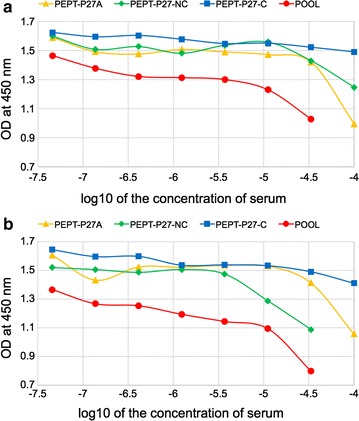
ELISA plates were coated with SAPN-Combo and antibody binding was inhibited by soluble antigen as indicates in the figure (**a** BALB/c; **b** C3H)

The immunogenicity of *SAPN*-*Combo* construct delivered in PBS buffer solution without addition of any adjuvant was tested in BALB/c and C3H mice. End point titers of about 1–2 × 10^6^ were obtained when ELISA plates were coated with the homologous construct (Table 2). Lower titers were obtained for coating with the peptides *Pept*-*P27A*, *Pept*-*P27*-*NC* and *Pept*-*P27*-*C* while the *Pept*-*P27*-*N* fragment is only weakly recognized. Since titers depend on the amount of each antigen bound to ELISA plates and its structural integrity, a better appreciation of the contribution of each antigen to the overall antibody response to *SAPN*-*Combo* is given by the determination of inhibition of antibody binding by single soluble antigens. Total inhibition is observed in the homologous mode (same coating and inhibitory antigen, data not shown) while partial inhibition (up to 50%) is observed when *SAPN*-*Combo* and single antigens (*Pept*-*P27A* and *Pept*-*P27*-*NC*) are used for coating and soluble inhibitors, respectively (Fig. 4). Much lower inhibition was observed for *Pept*-*P27*-*C* in line with the results obtained in ELISA where individual antigens were used to coat the plates (compare Table 2). A stronger inhibition is reached if all three antigens are used together as inhibitors.

**Table 2:** Serum titers of mice immunized with ***SAPN*** **-** ***Combo***

Coating antigen	BALB/c	C3H
*SAPN*-*Combo*	1.1 +/− 0 X 10^6^	2.0 ± 1.0 × 10^6^
Pept-P27A	8.3 ± 3.7 X 10^5^	1.3 ± 0.62 × 10^6^
Pept-P27-NC^a^	8.1 X 10^4^	2.4 × 10^5^
Pept-P27-C^a^	2.7 X 10^4^	8.4 × 10^4^
Pept-P27-N^a^	≤5.4 X 10^3^	≤5.4 × 10^3^

Sera were collected after the 3rd immunization. Titers of individual sera were only determined for SAPN-Combo and Pept-P27A

^a^Serum pools were used for the other individual peptides

## Discussion

Development of an effective asexual blood stage malarial vaccine candidate has been difficult and fraught with problems. Apparently, to generate the most effective candidate new antigen choices as well as new delivery technologies need to be developed. Here we demonstrated the addition of two new promising B-cell epitopes P27-NC and P27A from the Tex1 antigen to the SAPN delivery system. In particular, the P27A antigen is currently being evaluated in clinical trials as a synthetic peptide (ClinicalTrials.gov; PACTR201310000683408), thus improving its immunogenicity by coupling to a suitable carrier seems to be a highly promising approach.

**Fig. 5:**
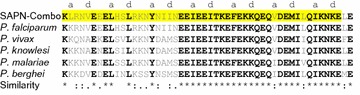
Sequence conservation of P27-N and P27-C in *SAPN*-*Combo* compared to other important *Plasmodium* strains multiple sequence alignment (*CLUSTAL O v1.2.4*). Completely conserved residues among all strains are shown in *black* and *bold*. The sequences are the following from *top* to *bottom*: XP_966024.1—*Plasmodium falciparum* 3D7; SCO68358.1—*Plasmodium vivax*; XP_002261647.1—*Plasmodium knowlesi*; SBS87522.1—*Plasmodium malariae*; XP_679242.1—*Plasmodium berghei* ANKA. The residues that are predicted to have a higher coiled-coil propensity than 0.9 for the *SAPN*-*Combo* are highlighted in *yellow* (*PCOILS v1.0.1*). The coiled-coil heptad-repeat with the core positions (**a**) and (**d**) is indicated above the sequences

The humoral immune response to the two segments of P27-NC clearly shows the relationship of immunodominant epitopes versus less immunogenic regions: The most prominently exposed portions of P27-NC, the C-terminal portion of the trimeric coiled coil (i.e. P27-C) induces a stronger immune response compared to the antibody production against the N-terminal region P27-N (Table 2), which is more buried in the core of the SAPNs (see Fig. 2d). Remarkably, for the corresponding shorter fragment *Pept*-*P27*-*C* 62% is recognized by 62% of donor’s sera from Burkina Faso (Table 1). Since the P27-C is also highly conserved among many different *Plasmodium* species (Fig. 5), this is a welcome feature of our SAPN design as it may induce cross-protection among different *Plasmodium* species, including the most prominent parasites species *P. falciparum* and *P. vivax*.

One of the major advantages of our SAPN technology is that it enables the addition of two B cell antigens, one on each terminus of the monomer to be displayed on the surface of the SAPNs. In addition, T-cell epitopes can be engineered into the SAPN core to be delivered to the antigen processing machinery of the cells. This unique design therefore allows for the inclusion of multiple T and B cell epitopes. In this case we included both P27-NC and P27A B-cell epitopes, which both have been established to be immunogenic in mice (P27 [10]), rabbits and humans (P27A [13], manuscript in preparation) in combination with a variety of CD4 and/or CD8 epitopes. Human purified antibodies specific for P27 and P27A have been shown to be active in ADCI [10, 14] and are associated with protection in people in endemic areas (unpublished results). By including both P27-NC and P27A antigens it is likely to have a higher probability of inducing protective antibodies in a population at large. The present design has the particular advantage of being very immunogenic without the need of any adjuvant.

In summary, these new SAPNs represent a new vaccine strategy. The epitopes P27 and P27A are expressed primarily on the asexual blood stage of the parasite’s life cycle, while CSP is expressed on the pre-erythrocytic stage. A CSP based vaccine will only prevent infection before clinical symptoms develop. An asexual blood stage vaccine will actually help deal with preventing parasitemia from further developing and reducing clinical symptoms. As both approaches continue to be developed they eventually can be admixed to generate a vaccine candidate that prevents the initial infection, and can eventually prevent any breakthrough infection.

## Conclusion

Here we have demonstrated that SAPNs induce an immune response akin to the one in seropositive individuals in Burkina Faso. The SAPNs described in this manuscript represent a key improvement over previous designs. Modification of the nanoparticles allowed P27 to be presented, which was not possible with the first designs of the SAPNs. Since P27 is highly conserved, this might even provide cross-protection among different *Plasmodium* species. If confirmed in human clinical trials, it would allow the development of a robust vaccine easy to manufacture, store and deliver to the needed population. These vaccine characteristics have been and remain the “holy grail” of vaccine development and formulation.
